# Role of thioredoxin reductase 1 in dysplastic transformation of human breast epithelial cells triggered by chronic oxidative stress

**DOI:** 10.1038/srep36860

**Published:** 2016-11-15

**Authors:** Chaoran Dong, Lei Zhang, Ruoxuan Sun, Jianying Liu, Hanwei Yin, Xiaoxiao Li, Xiaoqing Zheng, Huihui Zeng

**Affiliations:** 1 State Key Laboratory of Natural and Biomimetic Drugs, Peking University Health Science Center, Beijing 100191, P.R. China; 2 Department of Chemical Biology, School of Pharmaceutical Sciences, Peking University, Beijing 100191, P.R. China; 3 Department of Pathology, School of Basic Medical Sciences, Peking University, Beijing 100191, P.R. China; 4 Keaise Center for Clinical Laboratory, Wuhan 430000, P.R. China; 5 Department of pharmacy, Peking University Third Hospital, Beijing 100191, P.R. China

## Abstract

Thioredoxin reductase 1 (TrxR1) is a pivotal intracellular redox sensor and antioxidant enzyme. On the other hand, overexpression of TrxR1 is closely correlated with the initiation of various tumors including breast cancer, though the detailed mechanism remains unclear. Here we investigated the role of TrxR1 in dysplastic transformation of human breast epithelial cell line MCF-10A induced by chronic oxidative stress. Not surprisingly, sustained exposure to H_2_O_2_ significantly augmented the expression and activity of TrxR1 in MCF-10A cells. The dysplastically transformed MCF-10A (MCF-10AT) cells undergoing 8-week H_2_O_2_ treatment exhibited a certain degree of malignancy in tumorigenicity evaluation. Moreover, TrxR1 inhibitor ethaselen (BBSKE) could partially reverse some malignant phenotypes including epithelial to mesenchymal transition (EMT) of MCF-10AT as well as MCF-7 cells. Collectively, our results supported the considerable involvement of TrxR1 in the onset of breast cancer and BBSKE may be a promising agent against breast cancer.

Breast cancer is one of the leading malignancies in women around the world^1^. Enormous advances have been made in the field of breast cancer research over the past decades; however, it is still not fully understood about the detailed mechanism for mammary carcinogenesis. Estrogens are shown to initiate breast cancer by stimulating cell proliferation^2^, activating oncogenes^3^, inactivating tumor suppressor genes^4^,^5^ and causing oxidative DNA damages in an estrogen receptor (ER)-dependent and independent manner^6^. Moreover, the direct action of estrogen or its metabolites on cellular mitochondria can also generate excessive reactive oxygen species (ROS) accelerating the development and progression of breast cancer^7^. This off balance redox status of intracellular microenvironment is recognized as a pivotal phase in the process of mammary carcinogenesis as well as other tumorigenesis^8^.

It is generally known that there are several innate defense strategies (antioxidant enzymes, nonenzymatic antioxidants and physical barriers) aiming to overcome oxidative stress lesions. Nevertheless, overexpression or over-activation of certain antioxidant enzymes such as glutathione peroxidase and thioredoxin reductase (TrxR) in response to exceeding amount of ROS in turn might contribute to tumor development^9^. Given the special metabolism circumstance of transformed cells or cancer cells compared with normal cells, the deregulation of ROS scavengers can be viewed as pro-survival adaptive changes, which appears to echo the latest standpoint that cancer is an evolutionary product affected by dynamic tissue environment not only by oncogenic mutations^10^.

TrxR is a selenium-containing oxidoreductase that is responsible for catalyzing the NADPH-dependent reduction reaction of thioredoxin (Trx) disulfide and a broad spectrum of oxidized protein substrates^11^. TrxR is closely related with multiple cellular processes such as antioxidation defense, redox signaling, cell proliferation and apoptosis^12^,^13^. Mammalian TrxR consists of three isoforms: TrxR1 in the cytoplasm, TrxR2 in the mitochondria, TrxR3 primarily expressed in the testes^11^. Despite wide expression of TrxR1 in numerous types of tissue cells, higher levels of TrxR1 have been observed in various malignancies including non-small cell lung carcinoma and hepatocellular carcinoma than in normal tissues. In fact, it has been demonstrated that TrxR1 plays an important part in tumor growth, progression, metastasis, and chemotherapy resistance^14^,^15^. Therefore, TrxR1 has emerged as a promising biomarker and drug target for oncotherapy. Currently, a substantial body of small molecule inhibitors against TrxR1 has been identified to be potential anti-cancer agents such as metal containing compounds and natural products^16^,^17^,^18^,^19^. Nonetheless, the role of TrxR1 in the onset of breast cancer remains to be elucidated.

Although a great deal of studies utilizing estrogens or estrogenic chemicals to induce breast carcinogenesis has been previously established in cell models such as human mammary epithelial cell line MCF-10A, few studies about the direct ROS-triggered dysplastic or malignant transformation of MCF-10A cells, especially about the involvement of TrxR1 in this process have been reported yet. We hypothesized that persistent rising levels of intracellular ROS ultimately lead to mammary tumorigenesis and deregulation of TrxR1 probably participates in the promotion of breast cancer. In this work, long-term exposure to H_2_O_2_ in MCF-10A cells was employed to simulate the imbalanced redox context in the initial phase of breast tumor. We aimed to assess the influence of chronic oxidative stress on TrxR1 expression and activity in transformed MCF-10A cells. Besides, the relationship between alterations of cellular phenotype and TrxR1 during this transformation course was examined as well. TrxR1 might facilitate the occurrence of certain dysplastic phenotypes associated with breast cancer.

## Results

### Establishment of the cell model of mammary dysplasia

**Figure 1:**
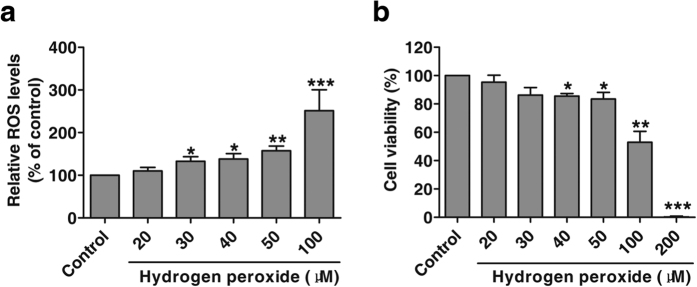
Determination of the proper concentration of H_2_O_2_ to induce chronic oxidative stress on MCF-10A cells. (**a**) Detection of ROS in MCF-10A cells after 24-h exposure to H_2_O_2_ (n = 4). Raw DCF fluorescence values were converted to relative DCF fluorescence, namely relative ROS levels expressed as fold over control (untreated cells). *P < 0.05, **P < 0.01, ***P < 0.001 versus control. (**b**) Alamar blue assay of viability of MCF-10A cells treated with H_2_O_2_ for 24 hours (n = 4). Data were means ± SD. *P < 0.05, **P < 0.01, ***P < 0.001 versus control (untreated cells).

To determine the appropriate concentration of H_2_O_2_ to induce the chronic oxidative stress in MCF-10A cells, we first examined intracellular ROS content in MCF-10A cells treated with H_2_O_2_ along with its effect on cell viability. As shown in Fig. 1a, the ROS level was induced by H_2_O_2_ (20 μM to 100 μM) in a dose-dependent manner after 24-h treatment. Accordingly, the viability of MCF-10A cells was gradually inhibited by increasing concentrations of H_2_O_2_ ranged from 20 μM to 200 μM (Fig. 1b). It should be noted that 30 μM H_2_O_2_ generated statistical upregulation of ROS in MCF-10A compared with control cells, whereas this dose of H_2_O_2_ had no significant cytotoxicity to MCF-10A cells yet. Based on these results, we selected 30 μM of H_2_O_2_ as the dosage and 8 weeks as the time range in the following experiments to establish the cell model of dysplastically transformed human breast epithelial cell (MCF-10AT).

### Morphological Changes of MCF-10A Cells Affected by Chronic Oxidative Stress

**Figure 2:**
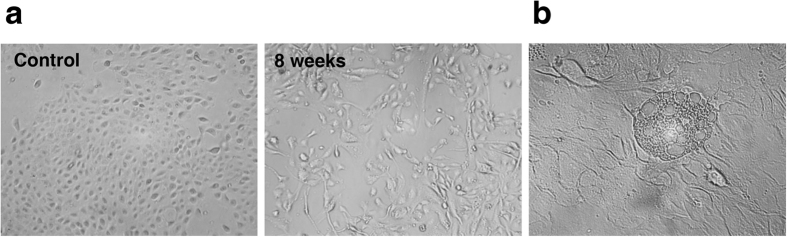
Morphological changes of MCF-10A cells under long-term H_2_O_2_ treatment. (**a**) Representative pictures of morphological variations of MCF-10A cells exposed to H_2_O_2_ for 0 and 8 weeks were photographed (10×). (**d**) Yellow arrow denotes the appearance of the focus formed by MCF-10A cells after 6-week H_2_O_2_ treatment.

MCF-10A cells were incubated with 30 μM H_2_O_2_ for long-term treatment. During this course, there were obvious morphological variations of MCF-10A cells that became larger and more extended. The typical pictures of MCF-10A cells treated with H_2_O_2_ for 8 weeks and untreated MCF-10A cells were shown in Fig. 2a. Moreover, we observed the appearance of focal changes of MCF-10A cells after 6-week continuous exposure to H_2_O_2_ (Fig. 2b), which seems to be the MCF-10A transformed foci associated with oxidative microenvironment described in previous study^20^.

### Characterization of Transformed MCF-10A Cells Induced by H_2_O_2_

**Figure 3:**
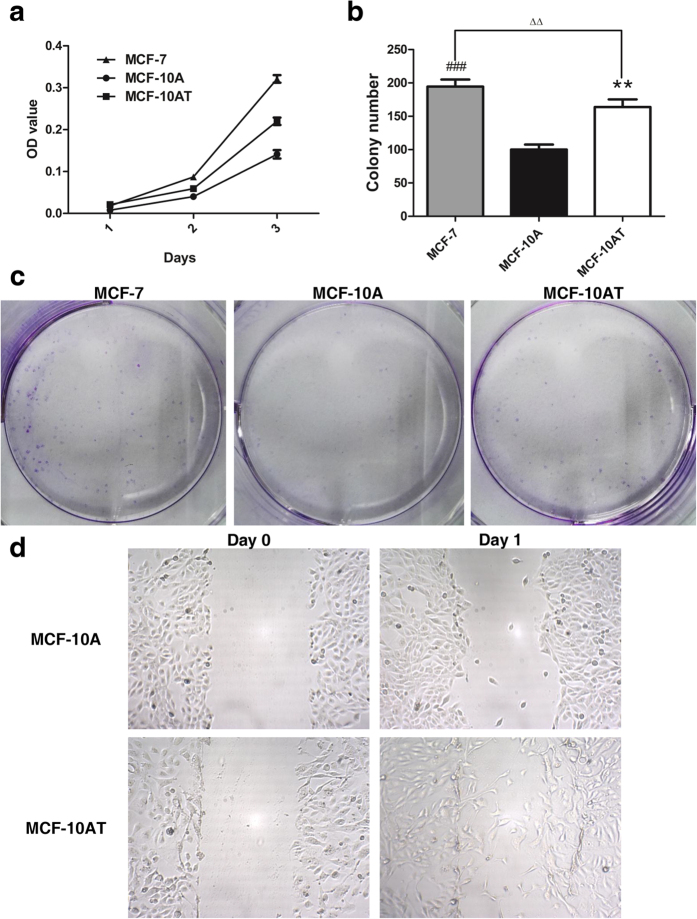
Effect of chronic oxidative stress on growth and migration potential of MCF-10A cells. Curve graph representation of cell growth in three days. (**b**,**c**) Clonogenic analysis of MCF-10A cells after 8-week H_2_O_2_ exposure. **P < 0.01, ^###^*P* < 0.001 versus MCF-10A cells. ^ΔΔ^*P* < 0.01 between MCF-10A cells after 8-week H_2_O_2_ treatment and MCF-7 cells. (**d**) Photomicrographs displaying increased migration ability of MCF-10A cells after chronic H_2_O_2_ treatment. Data were means ± SD.

**Figure 4:**
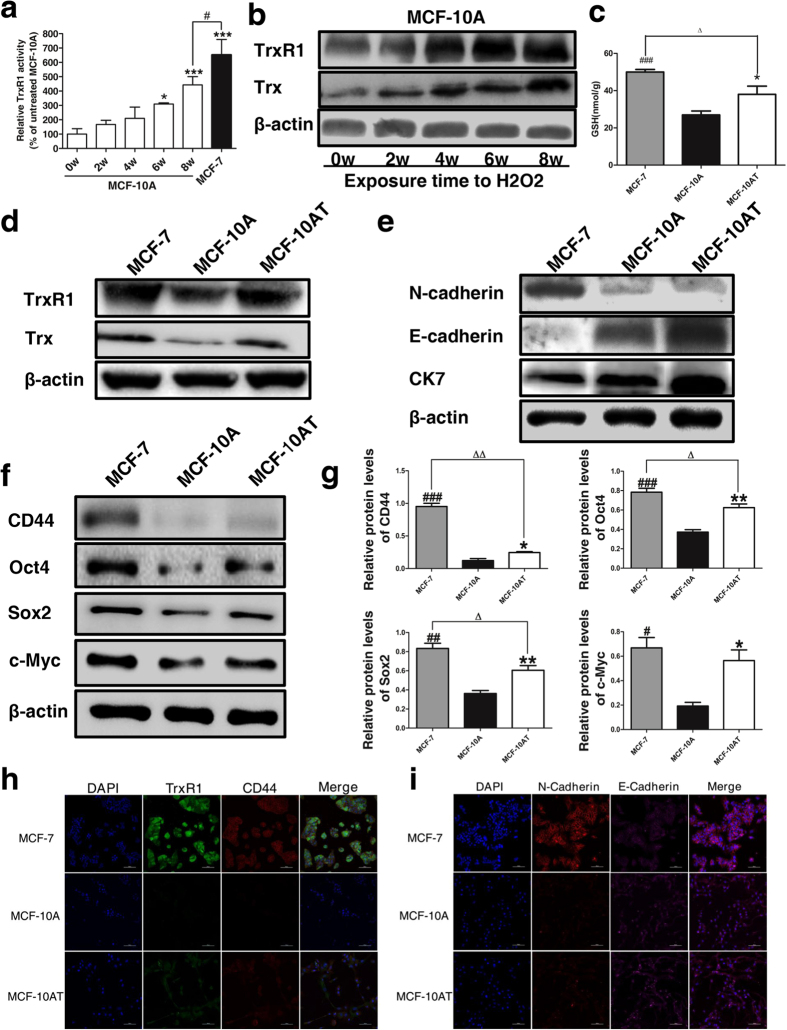
Characteristic analysis of transformed MCF-10A cells after H_2_O_2_ treatment. (**a**) TrxR1 activity assay of MCF-10A cells treated with H_2_O_2_ for 0, 2, 4, 6 and 8 weeks. MCF-7 cells were used as positive control. *P < 0.05, ***P < 0.001 versus untreated cells. ^#^*P* < 0.05 between MCF-10A cells after 8-week H_2_O_2_ treatment and MCF-7 cells. (**b**) Cropped western blot bands of TrxR1 and Trx in MCF-10A cells after exposure to H_2_O_2_ for 0, 2, 4, 6 and 8 weeks are presented here and all the blots have been run under the same experimental conditions. (**c**) Intracellular GSH level in MCF-7, MCF-10A and MCF-10AT cells. *P < 0.05, ^###^*P* < 0.001 versus MCF-10A cells. ^Δ^*P* < 0.05 between MCF-10A cells after 8-week H_2_O_2_ treatment and MCF-7 cells. (**d**) Western blot analysis of TrxR1 and Trx levels in MCF-7, MCF-10A and MCF-10AT cells. (**e**) Western blot analysis of N-Cadherin, E-Cadherin and CK-7 in MCF-7, MCF-10A and MCF-10AT cells. (**f**,**g**) Cropped western blot bands (**f**) and quantifications (**g**) of stem cell markers CD44, Oct4, Sox2 and c-Myc in MCF-7, MCF-10A and MCF-10AT cells. *P < 0.05, **P < 0.01 between MCF-10AT and MCF-10A, ^#^*P* < 0.05, ^##^*P* < 0.01, ^###^*P* < 0.001 between MCF-7 and MCF-10A, ^Δ^*P* < 0.05, ^ΔΔ^*P* < 0.01 between MCF-10AT and MCF-7. (**h,i**) Confocal immunofluorescence imaging of TrxR1 along with CD44 (**h**) and N-Cadherin along with E-Cadherin (**i**) in MCF-7, MCF-10A and MCF-10AT cells. Nuclei were counter-stained by DAPI. Scale bar, 100 μm. The representative images shown were from three independent experiments. Data were means ± SD. The uncropped western blotting images were displayed in Figure S1.

We firstly evaluated the impact of chronic oxidative stress towards the proliferative and migratory ability of MCF-10A. Alamar blue assay (Fig. 3a) and colony formation assay (Fig. 3b,c) revealed that long-term H_2_O_2_ exposure increased the growth and survival ability of MCF-10A cells. As displayed in Fig. 3d, H_2_O_2_ treatment effectively stimulated the migration of MCF-10A cells. TrxR1 is generally believed to serve as a redox sensor of intracellular environment^12^,^21^. The anaerobic metabolism along with unbalanced redox state under the hypoxia conditions in cancer cells tends to aggravate the deregulation of those pivotal antioxidant enzymes including TrxR1^14^,^15^, which was also validated in our current results. As displayed in Fig. 4a, the TrxR1 activity exhibited a time-dependent upregulation in MCF-10A cells upon prolonged exposure to H_2_O_2_ for 2, 4, 6 and 8 weeks, suggesting TrxR1 as one of ROS scavengers in response to chronic oxidative stimuli. Despite continuous elevation of TrxR1 activity in MCF-10A cells treated with H_2_O_2_, its level in MCF-10AT cells was still clearly lower than basal TrxR1 activity of breast cancer cell line MCF-7 (*P* < 0.05), which might reflect high-grade malignance of MCF-7 cells. In consistent with TrxR1 activity results, TrxR1 protein level in MCF-10A cells also rose along with their exposure time (2, 4, 6 and 8 weeks) to H_2_O_2_. Moreover, the protein level of Trx, the most important substrates catalyzed by TrxR, showed a similar uptrend with TrxR1 in H_2_O_2_-treated MCF-10A cells (Fig. 4b). It has been reported that Trx antioxidant pathways work synergistically with glutathione to initiate tumor progression^22^. Accordingly, the GSH level in MCF-10A cells were sharply elevated, but still lower than that in MCF-7 cells (Fig. 4c). Collectively, from the perspective of Trx/TrxR system, H_2_O_2_-treated MCF-10A cells seemed to be gifted with certain properties distinguished from untreated MCF-10A cells.

Then we analyzed a panel of cellular characteristics of MCF-10AT cells induced by H_2_O_2_. As shown in Fig. 4d, higher levels of TrxR1 and Trx were observed in MCF-10AT cells relative to MCF-10A cells. Furthermore, the expression of epithelial marker E-Cadherin and mesenchymal marker N-Cadherin was evaluated by western blot analysis. N-Cadherin protein level was much higher in MCF-7 cells in comparison with MCF-10A and MCF-10AT cells where there was very little expression of N-Cadherin (Fig. 4e), which was clearly verified under confocal microscopy (Fig. 4i). On the other hand, western blot results displayed that E-Cadherin protein level was far below in MCF-7 cells compared to MCF-10A and MCF-10AT cells, however, there was no visible difference of E-Cadherin expression among them in immunofluorescence results. Cytokeratin 7 (CK7), widely recognized as classical biomarkers for pathologic diagnosis of cancer^23^,^24^, were markedly higher in MCF-10AT cells (Fig. 4e). Unexpectedly, CK7 expression quantity appeared to be less in MCF-7 cells than in MCF-10AT cells. As displayed in Fig. 4f,g, H_2_O_2_ exposure enhanced the protein levels of stem cell markers CD44, Oct4, Sox2, c-Myc in MCF-10A cells in various degrees, indicating that long-term oxidative stress increased the stem-like properties of MCF-10A cells. While these protein levels in MCF-10AT cells were still lower than in MCF-7 cells except no statistical difference of c-Myc expression between the two cell lines (Fig. 4g). Immunofluorescence results also confirmed that TrxR1 and CD44 content of MCF-10A cells was clearly less than that of MCF-7 cells, and H_2_O_2_ treatment could increase the protein expression of them in MCF-10A cells (Fig. 4h). Collectively, the chronic oxidative stress promoted dysplastic transformation in MCF-10A cells through the acquisition of stem cell and EMT characteristics.

### Tumorigenicity Evaluation of MCF-10AT Cells in Nude Mice and human plasma TrxR1 activity assay

**Figure 5:**
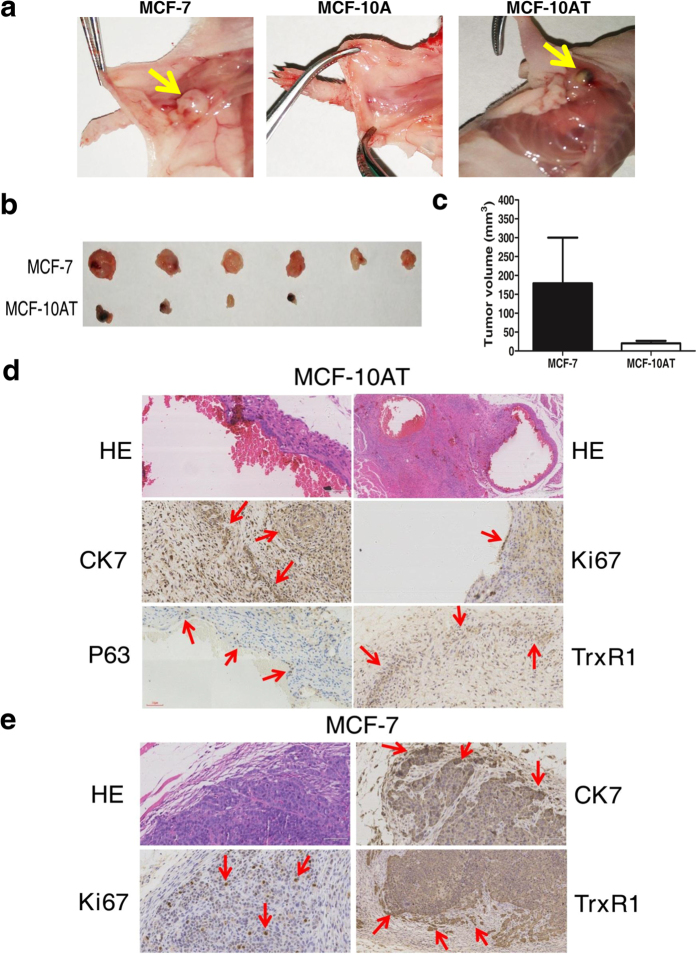
Tumorigenicity comparison between MCF-10AT and MCF-7 cells in nude mice. (**a**) Pictures of tumor sections from nude mice subcutaneously inoculated with MCF-7, MCF-10A and MCF-10AT cells. (**b**) Tumor or mass isolated from nude mice inoculated with MCF-7 and MCF-10AT cells. (**c**) Tumor volume of nude mice subcutaneously inoculated with MCF-7 or MCF-10AT cells. (**d**,**e**) Histopathology and immunohistochemical analysis of tumor sections from nude mice subcutaneously inoculated with MCF-10AT (**d**) or MCF-7 (**e**) cells. Data were means ± SD.

To further explore the features of MCF-10AT cells, xenograft tumor model was established by subcutaneously injecting MCF-10AT cells into athymic nude mice to evaluate their tumorigenicity. We observed that no mass or tumor could be observed in animals injected with MCF-10A cells. Meanwhile, four out of six (4/6) mice inoculated with MCF-10AT cells developed palpable mass in the injection site within one week. As positive control group, all the animals (6/6) injected with MCF-7 cells developed visible tumors within 3 days (Fig. 5a,b). Tumor volume of mice in positive control group was generally greater than those in animals injected with MCF-10AT cells (Fig. 5c).

Next, we performed histopathology and immunohistochemical analysis of tumor sections from above animals. HE staining results revealed that MCF-7 cells produced typical tumors in nude mice (Fig. 5e), while MCF-10AT cells formed breast epithelial hyperplasia and cysts in animals (Fig. 5d). Although MCF-10AT cells generated no obvious tumors in nude mice, they successfully survived and formed mammary gland ducts along with cyst-like structures, where these cells were positive for CK7 (Fig. 5d), suggesting MCF-10AT cells could maintain tumor-like growth property and present a certain degree of differentiation in nude mice. Likewise, CK7 was highly expressed in tumor tissues derived from MCF-7 cells exhibited epithelial feature (Fig. 5e). Besides, the proliferative rate was about 10–20% in MCF-10AT group assessed by Ki67 staining (Fig. 5d), while Ki67 was relatively higher expressed (20–30%) in the hotspot of tumor cells in MCF-7 group and a lot of mitotic figures were identified (Fig. 5e), indicating the active tumor proliferation. Additionally, P63 was expressed in part of the epithelial cells (MCF-10AT) of capsule wall (Fig. 5d), implying the existence of myoepithelial cells or intermediate type cells. Above all, high expression of TrxR1 was shown in tumor sections from both MCF-7 and MCF-10AT inoculated animals, though TrxR1 level was much higher in the former than in the latter (Fig. 5d,e). Moreover, there was a little reported phenomenon that frontier infiltration of TrxR1 was found in tumor tissues from MCF-7 cells (Fig. 5e), implicating that TrxR1 perhaps correlates with invasion and metastasis of breast cancer cells.

### Analysis of TrxR1 Activity in Human Plasma

In light of the potential of TrxR1 as a novel tumor biomarker, we compared the plasma TrxR1 activities of 241 female patients diagnosed as breast hyperplasia or breast cancer. According to the criteria published in 1990 [WHO Research on the menopause in the 1990s. World Health Organization Technical Report. 1996, 866], patients were divided into three age groups (age 35–44, 45–54 and 55+). Both breast hyperplasia and breast cancer group were similar for age distribution in each age group (*P* > 0.05), and there was no significant influence of age on TrxR1 activities in breast hyperplasia or breast cancer (*P* > 0.05). However, patients with breast cancer were reported to display a higher plasma TrxR1 activity compared with those with breast hyperplasia in all three age groups (Pre-menopause: *P* value = 0.0456, *Z* value = 0.02; Perimenopause: *P* value = 0.0168, *Z* value = 2.39; Post-menopause: *P* value = 0.0088, *Z* value = 2.62) (Table 1).

### Effect of TrxR1 inhibitor BBSKE on MCF-10AT and MCF-7 cells

**Figure 6:**
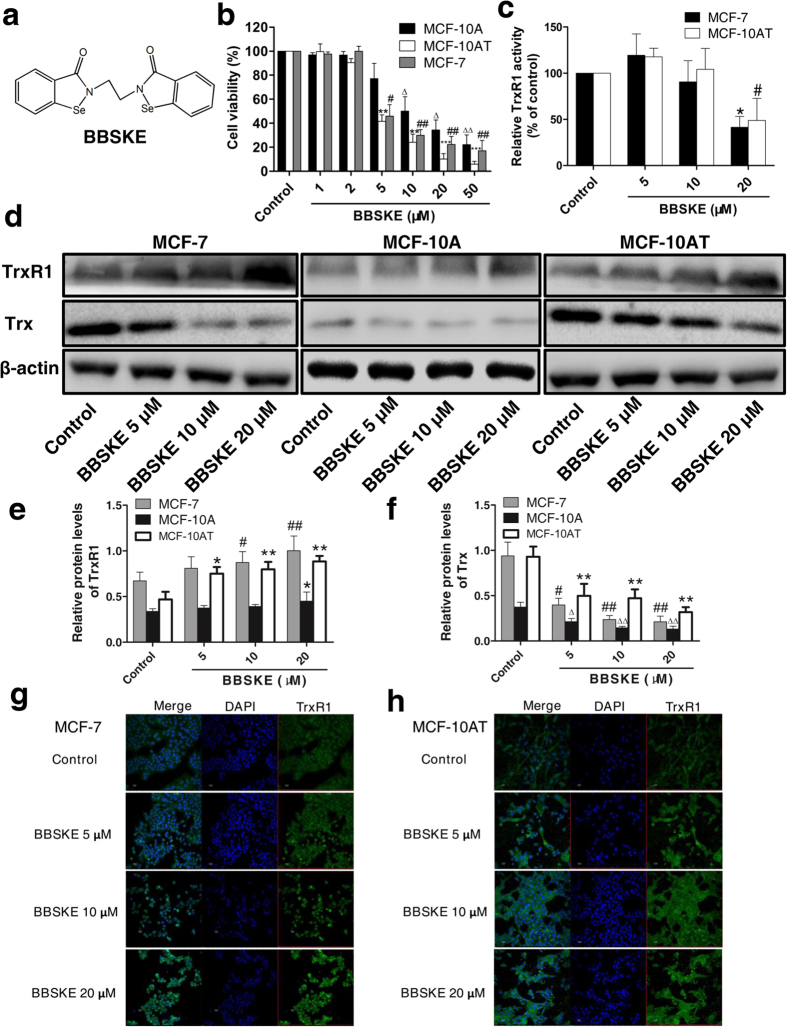
Enhanced inhibitory effect of TrxR1 inhibitor BBSKE on MCF-10AT and MCF-7 cells after 24-h treatment. (**a**) Chemical structure of TrxR1 inhibitor ethaselen (BBSKE). (**b**) Alamar blue assay of viability of MCF-10A, MCF-10AT and MCF-7 cells after 24-h BBSKE treatment (n = 4). **P < 0.01, ***P < 0.001, ^#^*P* < 0.05, ^##^*P* < 0.01, ^Δ^*P* < 0.05, ^ΔΔ^*P* < 0.01, compared with corresponding control cells. (**c**) TrxR1 activity assay of MCF-7 and MCF-10AT cells treated with BBSKE for 24 hours (n = 3). *P < 0.05, ^#^*P* < 0.05, compared with corresponding control cells. (**d**) Cropped western blot bands of TrxR1 and Trx in MCF-7, MCF-10A and MCF-10AT cells after 24-h exposure to BBSKE are presented here and all the blots have been run under the same experimental conditions. (**e**,**f**) Quantitation of protein levels of TrxR1 (**e**) and Trx (**f**) in MCF-7, MF-10A and MCF-10AT cells exposed to BBSKE for 24 hours (n = 4). *P < 0.05, **P < 0.01, ^#^*P* < 0.05, ^##^*P* < 0.01, compared with corresponding control cells. (**g,h**) Confocal immunofluorescence imaging of TrxR1 in MCF-7 (**g**) and MCF-10AT (**h**) cells after 24-h BBSKE treatment. Control group were untreated cells. Nuclei were counter-stained by DAPI. Scale bar, 100 μm. The representative images shown were from three independent experiments. Data were means ± SD. The uncropped western blotting images were displayed in Figure S2.

In consideration of the role of TrxR1 as a promising target for oncotherapy, it is reasonable to speculate that TrxR1 inhibitor may reverse dysplastic or malignant phenotype of cancer cells to some degree. The specific TrxR1 inhibitor ethaselen (1,2-[bis(1,2-benzisoselenazolone 3(2H)-ketone)] ethane, BBSKE) (Fig. 6a), a prospective anticancer agent developed by our laboratory^25^,^26^, was used to examine its effect on MCF-7, MCF-10 and MCF-10AT cells in the following experiments. Alamar blue assay results displayed that increasing concentrations of BBSKE (1, 2, 5, 10, 20 and 50 μM) reduced the viability of MCF-7, MCF-10AT and MCF-10A cells in a dose-dependent manner after 24-h treatment (Fig. 6b). Interestingly, the inhibitory effect of MCF-10AT and MCF-7 cells by BBSKE was obviously stronger than that of MCF-10A cells (Fig. 6b). The IC_50_ of BBSKE in MCF-7 and MCF-10AT cells after treatment for 24 hours was 4.1 μM and 3.9 μM respectively, while this value in MCF-10A cells was 7.8 μM, indicating that both MCF-7 and MCF-10AT cells were perhaps more sensitive to BBSKE treatment than MCF-10A cells. Besides, TrxR1 activity of both MCF-7 and MCF-10AT cells was sharply decreased by BBSKE treatment at 20 μM (*P* < 0.05) for 24 hours compared with corresponding control, although 5 μM BBSKE seemed to slightly augment TrxR1 activity (Fig. 6c).

**Figure 7:**
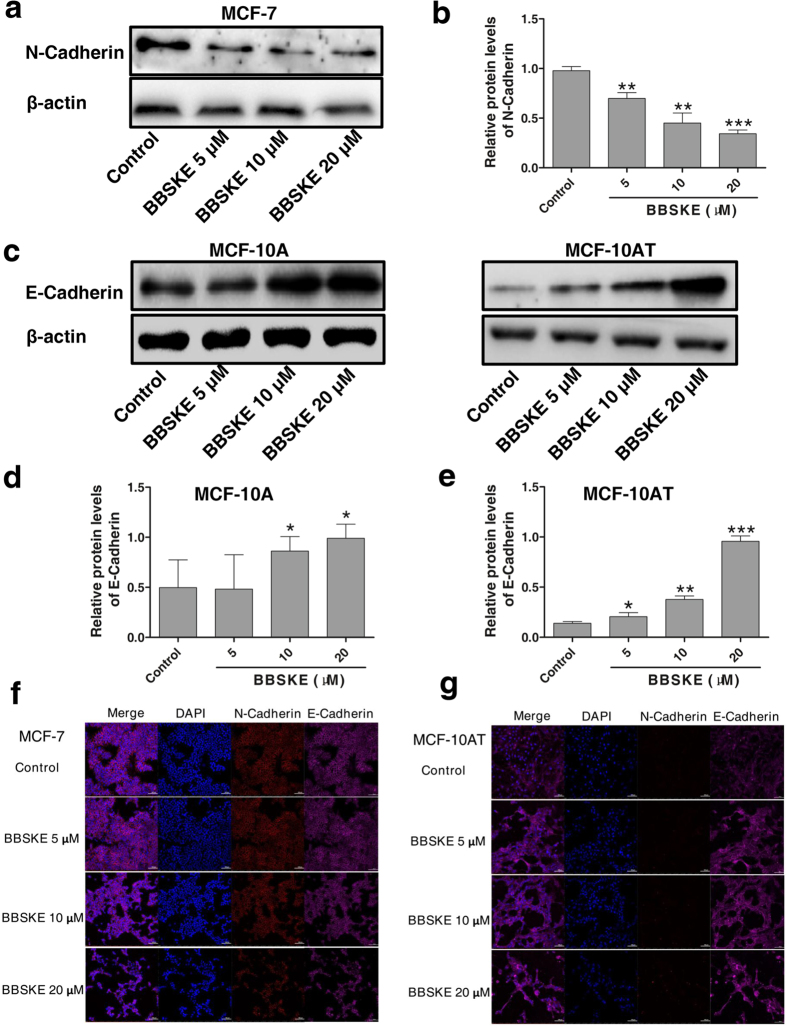
The impact of BBSKE on the protein expression of N-Cadherin and E-Cadherin in MCF-7 and MCF-10AT cells after 24-h treatment. (**a**–**e**) Quantitation of cropped western blot bands of N-Cadherin (**a**,**b**) in MCF-7 cells and E-Cadherin (**c**–**e**) in MCF-10A and MCF-10AT cells after 24-h BBSKE treatment (n = 4). All the blots presented here have been run under the same experimental conditions. *P < 0.05, **P < 0.01, ***P < 0.001 versus control cells. (**f**,**g**) Immunofluorescence stainings of N-Cadherin and E-Cadherin in MCF-7 (**f**) and MCF-10AT (**g**) cells treated with BBSKE (5, 10, 20 μM) for 24 hours were observed under confocal microscopy. Control group were untreated cells. Nuclei were counter-stained by DAPI. Scale bar, 100 μm. The representative images shown were from three independent experiments. Data were means ± SD. The uncropped western blotting images were displayed in Figure S2.

Contrary to the inhibition of TrxR1 activity by BBSKE in MCF-7 and MCF-10AT cells, TrxR1 protein levels in these two cell lines were raised by BBSKE treatment in a concentration dependent manner (Fig. 6d,e). There was a significant induction of TrxR1 expression by BBSKE compared with corresponding control in MCF-7 and MCF-10AT cells (Fig. 6d,e), which might be recognized as a negative feedback upregulation of protein expression owing to its activity inhibition and this phenomenon is similar to other protein enzymes as previously reported^27^. Additionally, Trx protein expression was markedly diminished by 5, 10, 20 μM BBSKE in these three cell lines after 24-h exposure (Fig. 6d,f). Confocal images of TrxR1 in MCF-7 and MCF-10AT cells treated with BBSKE were basically coincident with western blot results, though the changes of TrxR1 staining intensity were relatively less significant under microscopy (Fig. 6g,h). It should be noted that N-Cadherin protein level of MCF-7 cells exposed to BBSKE for 24 hours was prominently lowered (Fig. 7a,b,f) whereas there was an apparent elevation of E-Cadherin protein expression caused by BBSKE in MCF-10AT cells (Fig. 7c,e,g). The E-Cadherin level in MCF-10A cells was also observed enhanced while the increment was not as high as that in MCF-10AT cells (Fig. 7c,d). In the context of the relevance of N-Cadherin to invasion and metastasis of carcinoma cells and downregulation of E-Cadherin in malignant cells compared to normal cells^28^,^29^, it makes sense that TrxR1 inhibition by BBSKE may bring about reversion of some dysplastic phenotypes.

## Discussion

Breast dysplasia is a premalignant lesion associated with approximately fourfold increased risk of subsequent breast cancer^30^,^31^. In this study we investigated the role of TrxR1 in dysplastic transformation of normal human breast epithelial cell line MCF-10A caused by chronic oxidative stress, and offered evidence that deregulation of TrxR1 expression and activity may be associated with dysplastic phenotypes of MCF-10AT cells as well as breast cancer cell line MCF-7. Considering the stronger growth inhibition aroused by TrxR1 specific inhibitor BBSKE in MCF-10AT and MCF-7 cells rather than MCF-10A cells, we hold that TrxR1 is an underlying target for managing mammary carcinoma. BBSKE not only inhibited TrxR1 activity in MCF-7 and MCF-10AT cells but also decreased the expression of other tumor-related proteins (i.e. Trx, N-Cadherin) in MCF-7 cells and increased E-Cadherin protein level in MCF-10AT cells, further supporting TrxR1 involvement in the pathogenesis of breast cancer.

Overexpression of TrxR1, which has been described in various malignancies and cancer cells is thought to be closely associated with tumor growth promotion, progression and metastasis^32^,^33^,^34^. Our study revealed that the elevation of TrxR1 expression and activity was accompanied with dysplastic transformation of MCF-10A cells induced by long-term H_2_O_2_ exposure, suggesting that immoderate stimulation of TrxR1 machinery owing to chronic oxidative stress is perhaps a critical factor to trigger carcinogenesis in mammary epithelial cells. In spite of such relevance of TrxR1 to tumor development, it does not mean that this protein is consistently favorable to tumorigenesis. Carlson *et al.* reported that TrxR1-knockout mice subjected to chemical carcinogen exhibited a significant higher incidence of liver cancer than that of wild-type mice^35^, which is probably attributed to the antioxidation defense of TrxR1 to remove carcinogenic oxidants or repaired oxidized proteins. Thus, moderate TrxR1 level or excessive expression of TrxR1 may result in distinct outcome of normal cells. Besides, our finding implicates the potential use of TrxR1 as a screening biomarker for cancer prevention. As shown in Table 1, there was a significantly higher plasma TrxR1 activity in breast cancer patients than those with breast hyperplasia. Nonetheless, given the fact that TrxR1 expression or activity tends to be influenced by multiple pathological or physiological factors, it is necessary to perform integrative analysis of individual’s genetic, clinical and lifestyle factors so as to meet next-generation personalized medicine requirements as proposed in another research regarding biomarker variation and establishment of individualized cutoffs^36^.

Trx system exerts anti-oxidation protection, pro-survival effect and/or inhibition of apoptosis in normal cells and cancer cells^14^. However, unlike TrxR1, the role of Trx1 in carcinoma has been somewhat controversial. It was reported that Trx1-transgenic mice showed extended life span over control mice but without apparent signs of malignancy^37^, while Trx1 facilitated the invasion and metastasis of salivary adenoid cystic carcinoma (SACC) cells through mediating TGF-β-induced EMT in xenograft mouse model^38^. In our study, there was a significantly higher Trx protein level in MCF-7 cells than in MCF-10A cells where Trx expression was induced obviously by H_2_O_2_ treatment in a time-dependent manner. Moreover, in line with the upregulation of N-Cadherin and downregulation of E-Cadherin in SACC cells with Trx1 overexpression^38^, the similar expression profile of N-Cadherin and E-Cadherin was also observed in MCF-7 cells with higher expression of TrxR1 and Trx compared with MCF-10A cells. Interestingly, TrxR1 exhibited the feature of frontier infiltration in immunohistochemical analysis of tumor sections from MCF-7 inoculated animals (Fig. 5e). Previous research demonstrated that primary tumor cells undergoing EMT, namely losing expression of epithelial markers (e.g., E-Cadherin and EpCAM) and acquiring mesenchymal markers including N-Cadherin, tend to be more aggressive and apt to metastasis^28^,^29^. Therefore, it is of great possibility that TrxR1 drives the progression of breast cancer by catalyzing Trx1 along with its related EMT processes, though this hypothesis clearly needs more evidence to be supported. Stemness characteristics are also usually observed promoted in the process of dysplastic transformation and tumorigenic potential enhancement induced by H_2_O_2_^39^,^40^,^41^. In this study, four stem cell markers, (i.e. CD44, Oct4, Sox2 and c-Myc) were upregulated in MCF-10A cells after incubation with H_2_O_2_ for 8 weeks in accordance with the quantification results in Fig. 4g, which indicated that there could be a certain degree of dysplastic transformation in MCF-10AT cells, even if the differences appeared to be rather slight for CD44 and Sox2. Notably, the CD44 level was still far lower in MCF-10AT cells than MCF-7 cells, while the difference of c-Myc between the two cell lines was not statistically significant (*P* > 0.05).

Therapeutic implications of Trx system in cancer have been greatly studied, particularly with respect to the potential of TrxR1 as anti-cancer drug target^14^,^15^. Organoselenium compound BBSKE developed by our laboratory is a novel anticancer agent specifically inhibiting TrxR1 activity and is now under phase II clinical trial. There have been a series of reports regarding the antitumor efficacy of BBSKE alone or combined with other drugs against various malignancies *in vivo* and *in vitro*^42^,^43^,^44^, however, the effect of BBSKE on breast cancer cells is hardly known to date. Herein we observed that BBSKE significantly suppressed the growth of MCF-7 as well as MCF-10AT cells, whereas untreated MCF-10A cells were less susceptible to BBSKE treatment (Fig. 6b), suggesting the diverse sensitivity of antiproliferation effect of BBSKE between breast carcinoma cells and normal breast epithelial cells. In addition to the inhibition of TrxR1 activity, BBSKE sharply cut down the Trx protein expression in MCF-10AT and MCF-7 cells. Nevertheless, as mentioned above, TrxR1 inhibition may be just the reasonable response to the reduced Trx protein and not responsible for the decrease of total Trx protein amount. Thus there might be other unknown mechanisms accounting for this phenomenon. Another interesting observation from this study is that EMT was also markedly affected by BBSKE in MCF-10AT and MCF-7 cells. N-Cadherin of MCF-7 cells was significantly lessened and E-Cadherin in MCF-10AT cells was dramatically augmented under BBSKE treatment (Fig. 6d,e,f). These results are basically consistent with the impact of BBSKE on N-Cadherin/E-Cadherin protein levels in SACC cells^38^. In view of the close association between EMT and breast cancer metastasis^45^, BBSKE combined with other chemotherapeutics may be a promising strategy for reversing EMT and treating advanced breast malignancy.

In conclusion, our work demonstrates that chronic oxidative stress enables breast epithelial cells to undergo a certain degree of dysplastic transformation and deregulation of TrxR1 probably facilitate this pathological process, implicating the potential of TrxR1 as a diagnostic marker for breast cancer. Based on the susceptibility of MCF-7 and MCF-10AT cells to BBSKE, we can hypothesize that BBSKE or other kinds of TrxR1 inhibitors could be prospective chemotherapeutic agents for breast cancer treatment.

## Methods

### Reagents

Hydrogen peroxide (H_2_O_2_) solution (3%, w/v) was purchased from Sigma-Aldrich (St. Louis, MO, USA). Ethaselen (BBSKE) (PCT: CN02-00412) was synthesized in our research group (State Key Laboratory of Natural and Biomimetic Drugs, School of Pharmaceutical Sciences, Peking University, China) and dissolved in dimethyl sulfoxide (DMSO) to prepare a 20 mM stock solution. Both of them were freshly diluted by culture medium in all cell experiments. All other reagents utilized were acquired from standard commercial sources.

### Cell culture and treatments

Human breast cancer cell line MCF-7 and mammary epithelial cell line MCF-10A were obtained from cell resources center of Institute of Basic Medical Sciences, Chinese Academy of Medical Sciences (Beijing, China). MCF-7 cells were grown in Dulbecco’s modified Eagle’s medium (DMEM) (Macgene, Beijing, China) supplemented with 10% FBS (PAN Biotech, Aidenbach, Germany) and 1% antibiotic-antimycotic solution. MCF-10A cells were cultured in phenol red-free DMEM-F12 (Macgene, Beijing, China) medium supplemented with 5% horse serum (Invitrogen, Grand Island, NY, USA), epidermal growth factor (20 ng/ml), hydrocortisone (500 ng/ml), insulin (10 μg/ml), cholera toxin (100 ng/ml) and 1% antibiotic/antimycotic mixture. Both cells were maintained at 37 °C in a humidified incubator with 5% CO_2_. For chronic oxidative stress study, MCF-10A cells were exposed to H_2_O_2_ for 8 weeks, re-treated every 3 days and sub-cultured once a week.

### Cell viability assay

Alamar Blue assay was used to assess cell viability. Briefly, cells growing in 96-well plates were exposed to 100 μl culture medium containing no drug or different concentrations of H_2_O_2_ or BBSKE. After required time, 10 μl of Alamar Blue assay reagent (Pierce Biotechnology, Rockford, IL, USA) was added to each well and the plates were incubated at 37 °C in 5% CO_2_ for 2.5 hours. Absorption values at 570 and 630 nm was measured using a spectrophotometer Magellan F50 (Tecan, Männedorf, Switzerland) and OD_570_–OD_630_ was counted as the actual fluorescence intensity.

### ROS analysis

Intracellular ROS was detected with 2′,7′-dichlorofluoroscein diacetate (DCFH-DA) fluorescent probe (Applygen Technologies, Beijing, China). About 10^6^ cells in 6-well plates were incubated with 10 μM DCFH-DA at 37 °C in 5% CO_2_ for 30 minutes, and resuspended in 1 ml PBS. The fluorescence intensity of DCF was measured at 488 nm (excitation wavelength) and 525 nm (emission wavelength) by flow cytometer (BD FACSCalibur, Franklin Lakes, NJ, USA). Results were expressed as the mean fluorescence intensity of the analyzed cells.

### Cell morphology observation

The morphology of the MCF-10A cells after H_2_O_2_ treatment was observed by bright field imaging. Cells were treated with indicated concentration of H_2_O_2_ for 0 to 8 weeks in 6-well plates. At certain time point cells were washed with ice-cold PBS and observed under microscope (Olympus IX83, Japan).

### Western blot analysis

After indicated treatments, cells were lysed in RIPA buffer containing protease inhibitor. BCA Assay (Applygen Technologies, Beijing, China) was employed to determine protein concentrations. The protein samples were then electrophoretically separated by a 10% SDS-PAGE gel and transferred onto a polyvinylidene difluoride (PVDF) membrane. Then the membrane was blocked in 5% skimmed milk for 1 hour at room temperature. The desired primary antibodies of β-actin (ZSGB-Bio, 1:1000), TrxR1 (Abcam, 1:1800), CD44 (ABGENT, 1:1800), CK7 (Proteintech, 1:2000), E-Cadherin (BioLegend, 1:800), N-Cadherin (Biolegen, 1:800), Oct4 (Bioworld, 1:500), Sox2 (Bioworld, 1:1000), c-Myc (Bioworld, 1:500) and EGFR (ABGENT, 1:2000) were used to incubate with the membranes overnight at 4 °C. The membranes were then washed and incubated with horseradish peroxidase (HRP)-conjugated secondary antibodies (1:5000) for 1 hour at room temperature. Blots were visualized using the ECL kit (Advansta, Menlo Park, CA, USA). Band intensity was analyzed by Image Lab software, version 4.1 (Bio-Rad Laboratories) and β-actin was used as internal control for normalization.

### Confocal immunofluorescence imaging

Cells were seeded at a density of 2 × 10^4^/well on 4-well Lab-Teck II chamber slides (Nunc, Naperville, IL, USA). After indicated treatment, cells were washed twice with ice-cold PBS and fixed immediately with 4% paraformaldehyde in PBS for 15 minutes at room temperature. After two washes with PBS, cells were permeabilized with 0.25% Triton X-100 for 10 minutes at room temperature. Then cells were blocked with 1% bovine serum albumin (BSA) in PBS/Tween 20 for 30 minutes at room temperature and further incubated overnight with the primary antibodies against TrxR1 (Abcam, 1:300), CD44-PE (eBioscience, 1:160), E-Cadherin (BioLegend, 1:300) or N-Cadherin (Biolegend, 1:300) diluted in 1% BSA in PBS/Tween 20 in a humidified chamber at 4 °C. After three rinses with PBS, secondary antibodies conjugated with Dylight 488, Dylight 649 or Dylight 549 (Abbkine, 1:300) diluted in 1% BSA in PBS/Tween 20 (1:200) was added and incubated for 1 hour at room temperature in dark followed by three washes with PBS. Subsequently, nucleus were counterstained with 4′,6′-diamino-2-phenylindole (DAPI) for 10 minutes. Images were taken with a laser scanning confocal microscope (Nikon A1, Japan) and analyzed by NIS-Elements Viewer 4.20 software.

### Colony Formation Assay

To analyze the influence of chronic H_2_O_2_ treatment on colony formation rate, cells were seeded in 6-well plates (250 cells/well). After cultured in complete medium for 7 days, cells were fixed with methanol and stained by 0.1% crystal violet for 30 minutes. The number of colonies in each group were counted then.

### Wound healing analysis

Wound healing assay was performed to examine the effect of long term oxidative stress to the migration potential of MCF-10A cells. Cells were planted in 12-well at a density of 5 × 10^5^/well and grown to 80% to 90% confluence. A scratch were then made using a sterile pipette tip to create a cell-free area in each well and cell monolayers were washed with PBS to remove the cell debris. All cells were then culture in serum-free medium for another 48 h. The wound regions were observed and pictured under IX83 microscope.

### Determination of intracellular TrxR1 activity and GSH level

TrxR1 activity in cell extracts was determined under the DTNB (5,5′-dithiobis-2-nitrobenzoic acid) reduction assay. Different sources of cell lysates were collected with BCA assay used to quantify the protein concentration. A total volume of 80 μl reaction buffer (0.1 M PBS, 10 mM EDTA, 0.2 mg/ml BSA, pH 7.0–7.4) containing 50 μg protein was incubated with 20 μl 2 mM NADPH for 30 minutes at 37 °C in dark. 100 μl 10 mM DTNB was added to initiate the reaction afterwards. NADPH and DTNB were both dissolved in reaction buffer. The activity of TrxR1 was expressed by the linear increase rate of absorption values at 405 nm within the first 7.5 minutes. The intracellular GSH level was determined using GSH assay kit (Beyotime, Jiangsu, China) following the manufacturer’s instruction.

### Xenograft growth in nude mice

To evaluate the tumorigenicity of MCF-10A cells exposed to long term oxidative stress, xenograft model was made by subcutaneous injection of transformed MCF-10A (MCF-10AT) cells in athymic nude mice purchased from Laboratory Animal Department of Beijing University of Health Science Center. 4–5 week old female mice were randomized into three groups (6 mice in each group), which were inoculated subcutaneously in the right armpit with 100 μl PBS containing 10^7^ MCF-10A, MCF-10AT or MCF-7 cells (as a positive control), respectively. All the mice were kept in standard laboratory conditions and provided with *ad libitum* food and water. General health of these animals was daily observed and tumor growth at the injection site was monitored by palpation. Tumor volume were measured and calculated using the formula: length × (width)^2^ × 0.5. After one week, all mice were sacrificed by cervical dislocation and tumors were removed, weighed and fixed in 10% neutral-buffered formalin.

### Histopathology and immunohistochemical analysis

Tissues from nude mice were fixed in 10% buffered formalin and embedded in paraffin. Sections were dewaxed in xylene and gradually hydrated in graded ethanol. Antigen retrieval was achieved by microwaving in 0.01 M sodium citrate buffer (pH 6.0) for 20 minutes. After that, Slides were soaked in 3% hydrogen peroxide for 25 minutes to block the endogenous peroxidase followed by 30 minutes block with 3% BSA. Sections were incubated overnight at 4 °C with anti-human Ki67 antibody (Abcam, 1:500), anti-human TrxR1 antibody (Abcam, 1:100), anti-human CK7 antibody (Proteintech, 1:100), and anti-human P63 antibody (Abcam, 1:100). After washing in PBS, sections were incubated with two-step plus Poly-HRP anti-mouse/rabbit IgG detection reagents (KPL, 1:200) for 1 hour and exposed to DAB (DAKO) for 2 minutes. Sections were counterstained with hematoxylin for 3 minutes in, dehydrated in a graded alcohol solution, and mounted.

### Human Plasma TrxR1 activity analysis

A retrospective cross-sectional study based on clinical database of Keaise Clinical Examination Lab was performed in Wuhan, Hubei province, China between June 1st, 2015 and December 31th, 2015. The study analyzed the plasma TrxR1 activities of 241 female patients aged >34 years and diagnosed as breast hyperplasia or breast cancer. Human Plasma TrxR1 activity was determined by DTNB reduction assay as described before^46^. A unit of TrxR1 activity was expressed as 1 micromole of NADPH oxidized to NADP+ in one minute under assay conditions.

### Ethics statement

The study protocol involving human participants was approved by the Research Ethics Committee of Peking University. The methods were carried out in accordance with the approved guidelines. Informed consents were obtained from all patients.

The animal study protocol was approved by the Research Ethics Committee of Peking University and was conformed to the principles expressed in “Guide for the Care and Use of Laboratory Animals” (NIH, 1996). The methods were performed in accordance with “Regulations for the Administration of Affairs Concerning Experimental Animals” (Approved by State Science and Technology Commission, People’s Republic of China, 10/31/1988).

### Statistical Analyses

The statistical analyses of plasma TrxR1 activities between patients with breast cancer or breast hyperplasia were performed using Wilcoxon’s signed rank test. Significance values were calculated based on two-tailed tests, with *P* < 0.05 considered statistically significant. The analyses were performed with SAS software, version 9.3. For the other experiments, data collected from three or four independent experiments were presented as mean values ± standard deviation (SD) and analyzed using SPSS software, version 20.0. After the test of homogeneity of variances, statistical significance of mean values between multiple treatment groups (≥3) was determined by one-way ANOVA. Independent *t*-test (2-tailed) was performed to evaluate the difference between two groups. A *P* value < 0.05 was considered statistically significant.

## Additional Information

**How to cite this article**: Dong, C. *et al.* Role of thioredoxin reductase 1 in dysplastic transformation of human breast epithelial cells triggered by chronic oxidative stress. *Sci. Rep.* **6**, 36860; doi: 10.1038/srep36860 (2016).

**Publisher’s note:** Springer Nature remains neutral with regard to jurisdictional claims in published maps and institutional affiliations.

## Supplementary Material

Supplementary Information

## Figures and Tables

**Table 1:** Patients’ plasma TrxR1 activities by age group [*M (P*_25_, *P*_75_)].

Group	Breast hyperplasia			Breast cancer		
	*n*	Age (years)	TrxR1 (U/ml)	*n*	Age (years)	TrxR1 (U/ml)
Pre-menopause, age 35–44	82	39.5 (37, 42)	5.97 (4.33, 7.73)	11	41 (38, 43)	9.62 (5.72, 11.53)
Perimenopause, age 45–54	74	49 (46, 50)	6.28 (3.77, 8.12)	33	48 (47, 51)	10.87 (3.80, 19.87)
Post-menopause, age 55+	28	58.5 (56, 62)	5.61 (4.27, 7.01)	13	58 (56, 64)	11.37 (8.20, 14.20)
