# Supplementary material for: Role of thioredoxin reductase 1 in dysplastic transformation of human breast epithelial cells triggered by chronic oxidative stress
Source: Sci Rep. 2016 Nov 15;6:36860. doi: 10.1038/srep36860 (PMC5109291; doi:10.1038/srep36860)
Supplement: Supplementary Information [file srep36860-s1.pdf]

Role of thioredoxin reductase 1 in dysplastic transformation of human breast epithelial cells triggered by chronic oxidative stress

Chaoran Dong[1,2,+], Lei Zhang[1,2,+], Ruoxuan Sun[1,2], Jianying Liu[3], Hanwei Yin[4], Xiaoxiao Li[5], Xiaoqing Zheng[1,2], Huihui Zeng[1,2,*]

[1] State Key Laboratory of Natural and Biomimetic Drugs, Peking University Health Science Center, Beijing 100191, P.R. China

[2] Department of Chemical Biology, School of Pharmaceutical Sciences, Peking University, Beijing 100191, P.R. China

[3] Department of Pathology, School of Basic Medical Sciences, Peking University, Beijing 100191, P.R. China

[4] Keaise Center for Clinical Laboratory, Wuhan 430000, P.R. China

[5] Department of pharmacy, Peking University Third Hospital, Beijing 100191, P.R. China

[*] Corresponding. zenghh@bjmu.edu.cn

+these authors contributed equally to this work

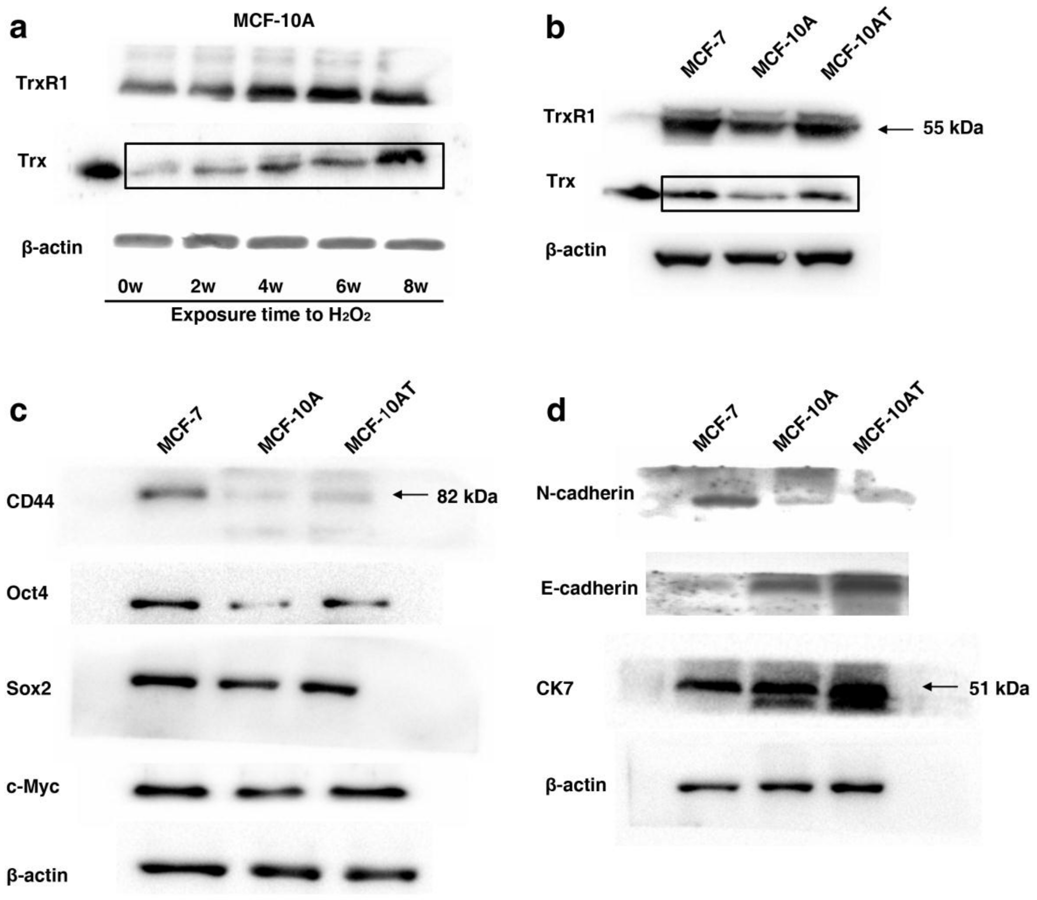

Figure S1. Uncropped images of western blot results

(a) Uncropped western blot bands of TrxR1 and Trx in MCF-10A cells after exposure to H_2O_2 for 0, 2, 4, 6 and 8 weeks. All the blots were obtained under the same experimental conditions. (b) Western blot analysis of TrxR1 and Trx levels in MCF-7, MCF-10A and MCF-10AT cells. (c) Western blot analysis of stem cell marker CD44, Oct4, Sox2 and c-Myc in MCF-7, MCF-10A and MCF-10AT cells. (d) Western blot analysis of N-Cadherin, E-Cadherin and CK-7 in MCF-7, MCF-10A and MCF-10AT cells. All results were optimized by the auto-exposure program of Image Lab software, version 4.1 (Bio-Rad Laboratories).

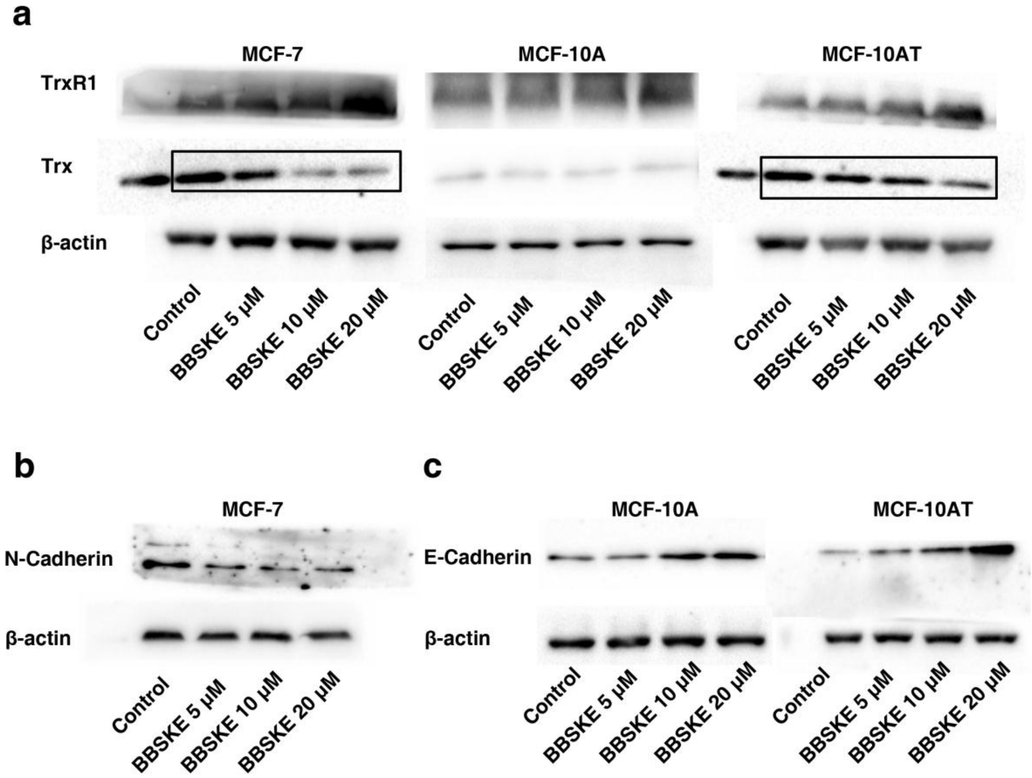

Figure S2. Uncropped images of western blot results

(a) Uncropped western blot bands of TrxR1 and Trx in MCF-7, MCF-10A and MCF-10AT cells after 24-h exposure to BBSKE. All the blots were obtained under the same experimental conditions. (b and c) Western blot analysis of N-Cadherin in MCF-7 cells and E-Cadherin in MCF-10A and MCF-10AT cells after 24-h BBSKE treatment (n=4). All results were optimized by the auto-exposure program of Image Lab software, version 4.1 (Bio-Rad Laboratories).
